# A survey of fever management in febrile intensive care patients without neurological injury

**DOI:** 10.1186/cc10387

**Published:** 2011-10-27

**Authors:** MK Saxena, NE Hammond, C Taylor, P Young, MC Reade, R Bellomo, J Myburgh

**Affiliations:** 1 The George Institute for Global Health, Sydney, NSW, Australia; 2 St George Hospital, Sydney, NSW, Australia; 3 St George Clinical School, University of New South Wales, Sydney, NSW, Australia; 4 Sydney Medical School, University of Sydney, NSW, Australia; 5 Medical Research Institute of New Zealand, Wellington, New Zealand; 6 Austin Hospital & University of Melbourne, VIC, Australia

## Introduction

Fever is a common observation during critical illness [1,2] and may be due to many possible causes such as infection, sterile inflammation and neurological injury. Clinical trials of fever management lack sufficient methodological quality to answer the question of whether attempts at reduction in temperature improves patient-centred outcomes in patients with sepsis, inflammation or neurological injury [3-7]. We undertook a survey to describe the attitudes of critical care clinicians in Australia and New Zealand towards fever management in critically ill patients without neurological injury or hyperthermic syndromes.

## Methods

An online scenario-based questionnaire survey was distributed to medical and nursing members of the Australian and New Zealand Intensive Care Society Clinical Trials Group (ANZICS-CTG) and their intensive care colleagues. Main outcome measures: the choice of drug and preferred threshold temperature for intervention with antipyretics in clinical practice and in a clinical trial.

## Results

**Figure 1:**
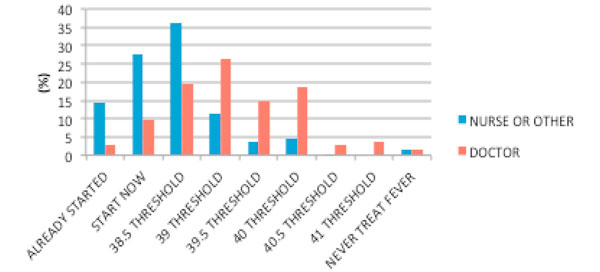
**Thresholds for initiation of antipyretic interventions between professions**. Nurse/other (*n *= 289); doctor (*n *= 134) (*P *< 0.0001).

**Figure 2:**
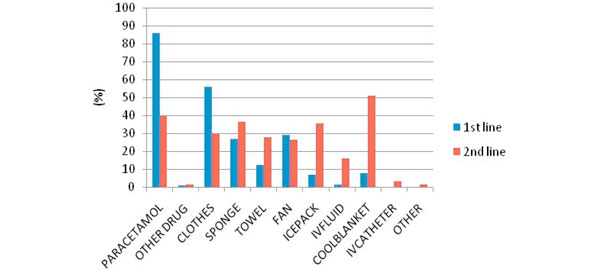
**First-line and second-line treatment preferences for fever management (*n *= 458)**.

There were 588 email invitations distributed through the ANZICS-CTG and Research Coordinator mailing list. Four hundred and forty-seven responses were received from 308 nurses (69%), 137 doctors (31%), and two others (0.5%). The majority of respondents having more than 8 years of experience (62%) worked in mixed medical and surgical units (84%) in a metropolitan or tertiary hospital setting (77%). The primary findings of our survey suggest that fever management is highly variable. Most clinicians administer an intervention to reduce temperature at or below 39°C (Figure 1); and initially use a combination of both pharmacological and physical interventions, with an increase in intensity of physical interventions for persistent fever (Figure 2). There were differences between the professions, with doctors choosing higher temperature thresholds for intervention and nurses generally using more physical cooling (Figure 1 and Table 1); fourthly, temperature thresholds for a clinical trial were 39.0°C (SD = 0.7°C) for a permissive strategy and 38.0°C (SD = 0.75°C) for an intensive strategy; finally, there was broad support for a clinical trial of fever management.

**Table 1:** Preference of first-line and second-line interventional category of antipyretic by profession

	First line (*n *= 418)			Second line (*n *= 409)		
	
	Nurse (%)	Doctor (%)	*P value*	Nurse (%)	Doctor (%)	*P value*
Pharmacological only	23	40	0.0002	5	5	0.87
Physical only	13	5	0.0087	58	62	0.36
Pharmacological and physical	64	55	0.077	38	33	0.39

## Conclusion

This survey suggests there is considerable clinical variability in fever management in patients with sepsis and without neurological injury or hyperthermic syndromes. At present, no particular management strategy is known to be superior to any other and it remains possible that current practice may be harming substantial numbers of patients. A temperature threshold of up to 40°C may be acceptable to clinicians for the design of a future randomized controlled trial. Further observational data may be informative for the design of such clinical trials.
